# The Antitumor Peptide ERα17p Exerts Anti-Hyperalgesic and Anti-Inflammatory Actions Through GPER in Mice

**DOI:** 10.3389/fendo.2021.578250

**Published:** 2021-03-17

**Authors:** Christophe Mallet, Ludivine Boudieu, Sylvain Lamoine, Catherine Coudert, Yves Jacquot, Alain Eschalier

**Affiliations:** ^1^ Université Clermont Auvergne, INSERM, NEURO-DOL Basics & Clinical Pharmacology of Pain, Clermont-Ferrand, France; ^2^ ANALGESIA Institute, Faculty of Medicine, Clermont-Ferrand, France; ^3^ Université de Paris, Faculté de Pharmacie de Paris, CiTCoM, CNRS UMR 8038, INSERM U1268, Paris, France

**Keywords:** GPER, ERα17p, pain, hyperalgesia, inflammation

## Abstract

Persistent inflammation and persistent pain are major medical, social and economic burdens. As such, related pharmacotherapy needs to be continuously improved. The peptide ERα17p, which originates from a part of the hinge region/AF2 domain of the human estrogen receptor α (ERα), exerts anti-proliferative effects in breast cancer cells through a mechanism involving the hepta-transmembrane G protein-coupled estrogen receptor (GPER). It is able to decrease the size of xenografted human breast tumors, in mice. As GPER has been reported to participate in pain and inflammation, we were interested in exploring the potential of ERα17p in this respect. We observed that the peptide promoted anti-hyperalgesic effects from 2.5 mg/kg in a chronic mice model of paw inflammation induced by the pro-inflammatory complete Freund’s adjuvant (CFA). This action was abrogated by the specific GPER antagonist G-15, leading to the conclusion that a GPER-dependent mechanism was involved. A systemic administration of a Cy5-labeled version of the peptide allowed its detection in both, the spinal cord and brain. However, ERα17p-induced anti-hyperalgesia was detected at the supraspinal level, exclusively. In the second part of the study, we have assessed the anti-inflammatory action of ERα17p in mice using a carrageenan-evoked hind-paw inflammation model. A systemic administration of ERα17p at a dose of 2.5 mg/kg was responsible for reduced paw swelling. Overall, our work strongly suggests that GPER inverse agonists, including ERα17p, could be used to control hyperalgesia and inflammation.

## Introduction

Estrogens and their classical receptors, *i.e.* ERα and β, interfere with pain pathways, through specific proteins and different molecular mechanisms (1). For example, 17β-estradiol (E_2_) facilitates heterodimerization of κ and µ opioid receptors *via* a membrane estrogen receptor (ER)-dependent process (1, 2). Opioid peptides exert antiestrogenic effects by interfering with AP-1-driven transcription (3). Thus, ERα and β could explain, at least in part, sex differences in pain sensitivity (1).

The newly discovered G protein-coupled estrogen receptor (GPER) is expressed, inter alia, in different regions of the central nervous system (CNS) such as the hippocampus and the hypothalamus, brain stem, the spinal cord, and autonomic and sensory ganglia (4–7), where it participates in a panel of neurophysiological events including pain. These effects are mediated through mechanisms involving an increase in the concentration of intracellular calcium and the accumulation of reactive oxygen species (ROS) (8–10). Likewise, the selective GPER agonist G-1 induces the depolarization of ventral and dorsal horn and cultured spinal neurons to mediate nociception, two events that are abolished by the specific GPER antagonist G-15 (5). Tamoxifen and fulvestrant, which also behave as GPER agonists, induce hyperalgesia (11–13). Hence, GPER has an indisputable role in nociception *via* rapid steroid hormone signaling pathways.

The 17-mer GPER-interacting peptide ERα17p (sequence: H_2_N-PLMIKRSKKNSLALSLT-COOH) was designed from the human ERα hinge and ligand-binding domains (residues 295-311) (14, 15). It corresponds to a surface-exposed polyproline II (PPII) region, which is composed of amino acids belonging to the C-terminus of the hinge region (D domain) and to the N-terminus of the AF2 transactivation function (E/F domains) (14). In the context of the whole protein, this fragment is in charge of the recruitment of transcription regulatory partners such as Ca^2+^-calmodulin (16) and Hsp70 (17). It is also subjected to post-translational modifications such as acetylation, phosphorylation, and SUMOylation [see (18) and references herein]. The KRSKK motif (residues 299–303), which is targeted by proteolytic enzymes (19), corresponds to the third ERα nuclear localization sequence (20). Hence, this part of the receptor appears crucial for the control of the turnover of ERα, its translocation and associated transcription.

In the light of the above observations, we have extensively studied the peptide ERα17p, notably in ERα-positive and -negative human breast cancer cells where it has been shown to exert a panel of activities. In steroid-deprived conditions, it promotes ER-dependent transcription and the proliferation exclusively of ERα-positive breast cancer cells through the activation of genes that are also activated by E_2_ (21–23). Thus, ERα17p can be seen as an estrogen-like molecule in these atypical experimental conditions. In breast cancer cells incubated in complete (physiological) culture medium, *i.e.*, in medium containing steroids and growth factors, it induces apoptosis (24). Since these effects are observed in both ERα-positive and -negative breast cancer cells with, however, a preference for ERα-positive cell lines, it is likely that a mechanism depending partially on ERα is involved (24). A decrease in the migration of breast cancer cells through actin cytoskeleton rearrangements is also observed (25). Accordingly, ERα17p decreases the size of tumors xenografted in mice by about 50%, at low dose (1.5 mg/kg) and over a short period (three times a week for 4 weeks) (24). These observations highlight the amazing pharmacological plasticity of G protein-coupled receptors (GPCRs) (26) and show the putative biased agonist character of ERα17p. ERα17p also induces the proteasome-dependent degradation of GPER and inhibits the activation of the epidermal growth factor receptor (EGFR) and of the extracellular signal-regulated kinase (ERK1/2). It also decreases the level of the protein c-fos (15). In combination with its GPER interaction, ERα17p interacts with artificial and breast cancer cell membranes (27, 28).

Because of the role of GPER in nociception (8, 29–33) and inflammation (34–42), it was decided to study the action of ERα17p on inflammation-induced hyperalgesia and edema, by using complete Freund’s adjuvant (CFA) and carrageenan mice models, respectively. The involvement of ERα17p in hyperalgesia and inflammation was evaluated by testing its action *in vivo*, in the presence and in the absence of G-15, a selective GPER antagonist. Strikingly, systemically administered ERα17p supports anti-nociception between 2.5 and 10 mg/kg, a dose range for which an antitumor activity, against ERα-negative breast tumors, has previously been observed, *in vivo* (24). Thus, targeting the GPER could be a promising approach not only to fight cancer, but also to control inflammation and related pain. Therefore, ERα17p could be proposed as a lead compound for the synthesis of new a generation of polymodal (antitumor, analgesic, and anti-inflammatory) drugs.

## Material and Methods

### Animals

Male mice CD1 (20–22 g, Janvier, France) were acclimatized for a week before testing. They were housed under controlled environmental conditions (21–22°C; 55% humidity, 12 h light/dark cycles, food and water *ad libitum*). Male and female mice CD1 have been used for the fluorecent imaging experiment.

### Ethics

The studies involving animals were reviewed and approved by the Auvergne Animal Experiment Ethics Committee, CE2A, and by the French Ministry of Higher Education and Innovation (authorization N° 18022) and performed according to European legislation (Directive 2010/63/EU) on the protection of animals used for scientific purposes, and complied with the recommendations of the International Association for the Study of Pain (IASP).

### Chemicals

The selective GPER antagonist G-15 [(3aS*,4R*,9bR*)-4-(6-bromo-1,3-benzodioxol-5-yl)-3a,4,5,9b-3*H*-cyclopenta[*c*]quinoline] was purchased from Tocris Bio-Techne SAS (Noyal-Châtillon-sur-Seiche, France). Morphine and λ-carrageenan were purchased from Sigma Aldrich (Saint-Quentin-Fallavier, France). We used the Fmoc strategy to synthesize the peptide ERα17p (sequence: H_2_N-PLMIKRSKKNSLALSLT-COOH) and its Cy5-labeled analogue [sequence: H_2_N-ERα17p-Pra(Cy5)-COOH], as previously described (15, 27). Briefly, the Cy5-labeled peptide was obtained by adding a propargylglycine (Pra) in the C-terminus of ERα17p and then, the Cy5 fluorescent probe on the propargyl moiety by using the click chemistry strategy (15). Then, the peptides were purified by reverse phase HPLC and identified by MALDI-TOF mass spectrometry (15).

All tested molecules were dissolved in saline solution except for G-15 which was dissolved in saline with 5% Tween80 and 5% DMSO. Drug solutions were prepared extemporaneously before use.

Complete Freund’s adjuvant (CFA), which was administered by periarticular injection, consists of *Mycobacterium butyricum* (Ref DF0640-33-7, Difco Laboratories, Detroit, USA) dissolved in paraffin oil and aqueous saline solution (0.9% NaCl) prior to an autoclave sterilization for 20 min at 120°C.

### Intracerebroventricular and Intrathecal Injections

Injections were carried out in mice anaesthetized with isoflurane (1–2%). Intracerebroventricular (i.c.v.) injections were made at the bregma level with a syringe and a calibrated needle with a guide so that the needle length was 4 mm (43). The injected volume was 2 µl *per* mouse. For intrathecal (i.t.) injections, the anesthetized mouse was held in one hand by the pelvic girdle and a 25-gauge × 1-inch needle connected to a 25 µl Hamilton syringe was inserted into the subarachnoid space between lumbar vertebrae 5 and 6 until a tail flick was elicited (44). The syringe was held in position for a few seconds after the injection of a volume of 2 µl *per* mouse.

### Monoarthritic Model

A persistent inflammatory pain model was produced by injection, under brief anesthesia (2.5% isoflurane inhalation), of 5 µl of CFA on either side of the left ankle joint of male mice (45). Behaviors tests were performed before and 7 days after CFA injection.

### Von Frey Test

Mice were acclimatized to the testing environment before baseline testing. The experimenter was blinded to the mice treatments. On the behavior testing day (7 days after CFA injection), mice were placed individually in Plexiglas compartments 8 cm (L) × 3.5 cm (W) × 8 cm (D), on an elevated wire mesh platform to afford access to the ventral surface of the hindpaws and were allowed to acclimatize for 1 h before testing. Von Frey filaments ranging from 0.02 to 1.4 g were applied perpendicularly to the plantar surface of the paw. Paw withdrawal or licking was considered as a positive response. Fifty percent paw withdrawal threshold (PWT) in grams was determined with a modified version of the Dixon up–down method, as previously described (46).

### Carrageenan Model and Edema Measurement

Paw edema was induced in male mice by an intraplantar (left hindpaw) subcutaneous injection of 20 µl of 3% λ-carrageenan with a 50 µl Hamilton syringe and a 26-gauge needle (43). Paw edema was measured before induction of inflammation and the effects of the drugs were assessed 4 h after carrageenan injection with a caliper.

### *Ex Vivo* Fluorescence Imaging

*Ex vivo* fluorescence imaging was performed with the IVIS Spectrum system (Perkin Elmer, Waltham, MA, USA) and a Cy5 filter set (excitation wavelength: 640 nm; emission wavelength: 680 nm). The peptide H_2_N-ERα17p-Pra(Cy5)-COOH (2 mg/kg) was injected intraperitoneally to female and male mice that were sacrificed 30 min post-injection. The brain and spinal cord were then removed to perform *ex vivo* fluorescence imaging of isolated organs. All images were acquired and analyzed with Living Image 4.7.2 software (PerkinElmer, Waltham, MA, USA). Experiments were performed on the IVIA multimodal imaging platform (Clermont-Ferrand, France).

### Experimental Protocol

The design, analysis and reporting of the research were carried out in accordance with the ARRIVE guidelines (47). Treatments were administered according to the method of equal blocks, in order to assess the effect of the different treatments over the same time interval, thereby avoiding unverifiable and time-variable environmental influences. All behavioral tests were performed in a quiet room by the same blinded experimenter. To ensure the methodological quality of the study, we followed the recommendations of Rice et al (48). Intraperitoneal (i.p.) administrations of ERα17p (1.25, 2.5, and 10 mg/kg), morphine (1 mg/kg), H_2_N-ERα17p-Pra(Cy5)-COOH (2 mg/kg), and G-15 (0.3 mg/kg) were performed with a constant volume of 10 ml/kg. To investigate the influence of GPER in the response to ERα17p, the selective GPER antagonist G-15 was administrated either i.p. (0.3 mg/kg, 10 ml/kg), i.c.v. (5 µg/mouse in 2 µl), or i.t. (5 µg/mouse in 2 µl) 20 min before ERα17p. The local anti-inflammatory effect of the peptide was investigated by an intraplantar (i.pl.) injection of ERα17p (20 µg in 10 µl).

### Statistical Analysis

Results were expressed as mean ± SEM and were recorded with Prism 7 (GraphPad™ Software Inc., San Diego, CA, USA). Data were tested for normality (Shapiro-Wilk test) and for equal variance (Fisher test). Multiple measurements were compared with two-way ANOVA. For kinetic data, the *post hoc* comparisons were performed by the Sidak test (number of groups = 2) or by the Dunnett test (number of groups > 2). The Kruskal-Wallis *post hoc* test was performed to have a mean comparison of the area under the time-course curves (AUC). Values of *p* < 0.05 were considered statistically significant. The AUC (0–180 min.) of 50% mechanical threshold (individual values) were calculated by the trapezoidal rule taking in reference the PWT baseline after CFA (threshold at time T_0_). The AUC of individual values is the sum of each area between experimental times from 0 to 180 min. calculated as: (time T − time before time T) × [(threshold at time T − threshold at time T_0_) + (thresholds obtained at time T_0_ or at time before time T − threshold at time T_0_)/2]. AUC was expressed as mean ± SEM (in g × min.).

## Results

### ERα17p Reduces Hyperalgesia

**Figure 1:**
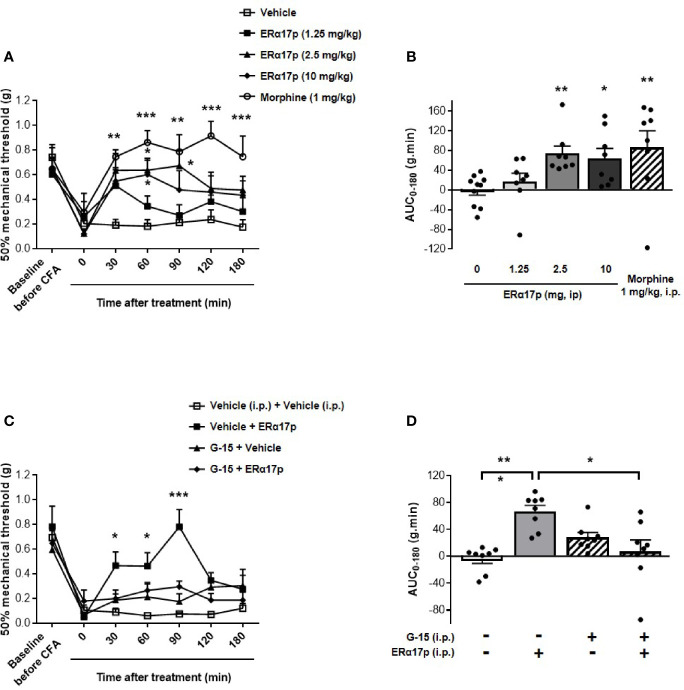
GPER-dependent action of ERα17p in tactile hypersensitivity in a CFA model. The Von Frey test was performed to assess the impact of ERα17p on CFA-induced mechanical hypersensitivity in inflammatory pain. The 50% paw withdrawal threshold (PWT) was determined with a modified version of the Dixon up–down method. **(A)** The anti-hyperalgesic action of ERα17p was determined by measuring dose-dependent effects. The Von Frey test was assessed before injection of CFA (baseline) and after that of vehicle (saline solution) or ERα17p (1.25, 2.5, and 10 mg/kg, i.p.) 7 days after CFA injection. **(C)** Involvement of GPER was determined using ERα17p with or without G-15. Mice were i.p. pre-treated with vehicle (5% DMSO, 5% Tween80 in saline solution, reference) or G-15 (0.3 mg/kg) 15 min before administration of vehicle (saline) or ERα17p (2.5 mg/kg, i.p.). **(B, D)** Area under the time-course AUC (0–180 min) of PWT variations obtained from **(A, C)**, respectively. Data are expressed as mean ± SEM (n = 8–10 *per* group). *p < 0.05, **p < 0.005, ***p < 0.001, when compared to the vehicle group (or G-15+ERα17p group, as mentioned in **D**); two-way ANOVA followed by Dunnett *post hoc* test for time comparison or Kruskal-Wallis test for AUC mean comparison.

To explore the action of ERα17p on hyperalgesia, we used the von Frey test in a complete Freund’s adjuvant (CFA) model (arthritis model). A decrease in the mechanical paw withdrawal threshold (PWT) was observed from 0.66 ± 0.05 g to 0.20 ± 0.04 g (n = 42, *p* < 0.001, *t*-test) for all mice, 7 days after CFA injection (**Figure 1A**). A PWT value of ~0.20 g was recorded with the vehicle (control, saline solution at 10 ml/kg) throughout the experiment. At 30 min and at a dose of 1.25 mg/kg i.p., the peptide induced a transitory anti-hyperalgesic effect. A marked decrease in hyperalgesia was observed at higher doses, *i.e.*, between 2.5 and 10 mg/kg i.p., from 30 to 90 min (**Figure 1A**). The values obtained for 60 min were: 0.64 ± 0.10 g for ERα17p at 2.5 mg/kg and 0.60 ± 0.12 g for ERα17p at 10 mg/kg (control: 0.18 ± 0.05 g, *p* = 0.04, Dunnett *post-hoc* test). These results were confirmed by calculation of the area under the curve (AUC, in g.min.), where a significant difference was observed between ERα17p (2.5 and 10 mg/kg) or morphine (1 mg/kg, i.p., used as positive control) treated mice and vehicle-treated mice. The AUC values recorded for 2.5, 10 mg/kg ERα17p and morphine were 74.2 ± 15.3 g.min. (*p* = 0.006), 64.6 ± 19.8 g.min. (*p* = 0.046), and 86.6 ± 33.7 g.min. (*p* = 0.003), respectively (Kruskal-Wallis test; AUC vehicle: −0.38 ± 9.95 g.min., **Figure 1B**).

These results were confirmed in a standard screening test used for analgesic candidates with acetic acid-induced inflammation. The two previous most active doses of ERα17p were tested in mice after an intraperitoneal injection of acetic acid 0.6% i.p. At the doses of 2.5 and 10 mg/kg, a significant decrease in the number of abdominal writhings was observed (2.86 ± 2.32, *p* < 0.001 and 8.13 ± 4.62, *p* = 0.017, respectively; vehicle: 30.63 ± 4.22, Kruskal-Wallis test, **Supplementary Figure 1A**). ERα17p at a dose >10 mg/kg failed to modify spontaneous locomotor activity (**Supplementary Figure 1B**).

### The Anti-Hyperalgesic Action of ERα17p Is GPER-Dependent

In the second part of this work, we studied the involvement of GPER in the anti-hyperalgesic action of ERα17p. The peptide used at 2.5 mg/kg i.p. was administered to CFA mice 30 min after an injection of the specific GPER antagonist G-15 (0.3 mg/kg i.p.) (49). As previously observed, ERα17p significantly increased PWT at 30 min (ERα17p: 0.47 ± 0.11 g; vehicle: 0.09 ± 0.03 g, *p* = 0.04, Dunnett *post-hoc* test), 60 min (ERα17p: 0.46 ± 0.11 g; vehicle: 0.06 ± 0.01 g, *p* = 0.02, Dunnett *post-hoc* test), and 90 min (ERα17p: 0.78 ± 0.11 g; vehicle: 0.08 ± 0.01 g, *p* < 0.001, Dunnett *post-hoc* test). G-15, inactive by itself, abolished the anti-hyperalgesic action of ERα17p (**Figure 1C**). These results were confirmed by AUC values over 180 min (ERα17p: 66.9 ± 9.0 g.min. *vs* ERα17p + G-15: 7.2 ± 17.2 g.min., *p* = 0.018 and AUC vehicle: −3.7 ± 6.7 g.min. *vs* ERα17p + G15, *p* < 0.9, Kruskal-Wallis test), as shown in the **Figure 1D**.

### ERα17p Diffuses Into the Brain and Spinal Cord

**Figure 2:**
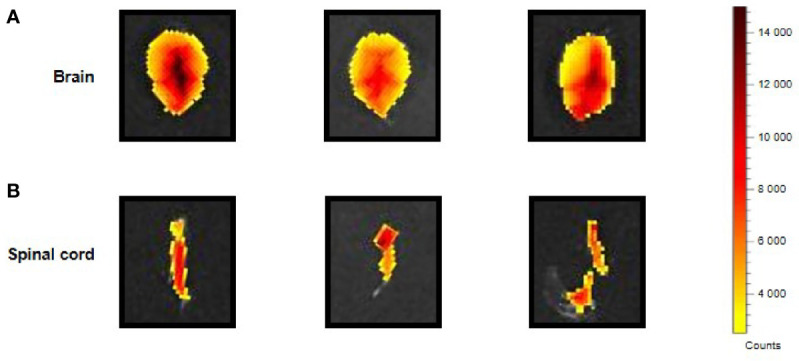
CNS distribution of the Cy5-labeled ERα17p peptide. Upper views of brain **(A)** and spinal cord **(B)** sampled from three mice 30 min after an i.p. injection of H_2_N-ERα17p-Pra(Cy5)-COOH (2 mg/kg).

The i.p. injected ERα17p distribution in the CNS was determined by using a Cy5-labeled version of the peptide [*i.e.*, H_2_N-ERα17p-Pra(Cy5)-COOH], which we used in a previous work (15). *Ex vivo* fluorescence staining showed an important diffusion of the Cys-5-labeled peptide in the supra-spinal (**Figure 2A**) and spinal (**Figure 2B**) compartments.

### Only the Supraspinal Pool of GPER Is Involved in the Anti-Hyperalgesic Action of ERα17p

To determine the site of the central action of ERα17p, two series of experiments were performed: assessment of its effect 1) after its central injections and 2) after its systemic injection, following a central administration of the GPER antagonist G-15.

**Figure 3:**
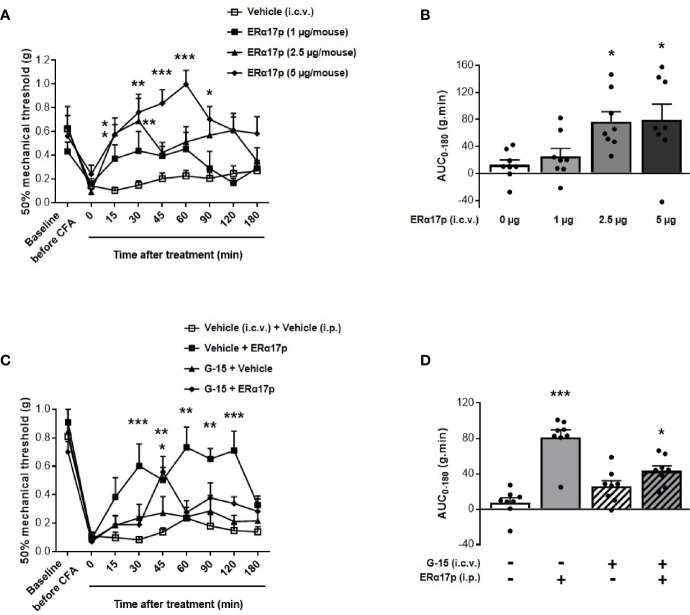
Involvement of supraspinal GPER in ERα17p action in the CFA model. **(A, C)** Area under the time-course AUC (0–180 min) of PWT variations from **(B, D)**, respectively. **(B)** Time-course effect of an i.c.v. administration of vehicle (saline solution, 2 µl/mice) or ERα17p (1, 2.5 and 5 µg/mice) on mechanical hypersensitivity in CFA mice model. **(D)** Involvement of supraspinal GPER in the action of ERα17p with or without G-15 i.c.v. Mice were i.c.v. pre-treated with vehicle (5% DMSO, 5% Tween80 in saline solution, 2 µl/mice) or G-15 (5 µg/mice) 20 min before administration of vehicle (saline solution, reference) or ERα17p (2.5 mg/kg, i.p.). Data are expressed as mean ± SEM (n = 8–9 *per* group). *p < 0.05, **p < 0.01, ***p < 0.001 compared with the vehicle group; two-way ANOVA followed by Dunnett *post hoc* test for time comparison or Kruskal-Wallis test for AUC mean comparison.

An intracerebroventricular injection of 1 µg/mouse of the peptide failed to induce a significant increase of thresholds. In contrast, a significant enhancement of PWT was observed 15 and 30 min after an i.c.v. injection of 2.5 µg/mouse of ERα17p (*p* = 0.04 and *p* = 0.01, respectively, Dunnett *post-hoc* test, **Figure 3A**). With 5 µg/mouse, a more robust anti-hyperalgesic effect was detected from 15 to 90 min, with a maximum at 60 min (**Figure 3A**). Assessment of AUC confirmed this dose-dependent effect: a dose of 1 µg/mouse failed to induce any change in PWT (AUC ERα17p 1 µg/mouse: 25.38 ± 11.7 g.min.; AUC vehicle: 12.47 ± 7.66 g.min., *p* > 0.9, Kruskal-Wallis test, **Figure 3B**). Significant effects were recorded with 2.5 µg/mouse ERα17p (AUC: 76.45 ± 17.79 g.min., *p* = 0.011, Kruskal-Wallis test) and 5 µg/mouse (AUC: 79.49 ± 22.98 g.min., *p* = 0.012, Kruskal-Wallis test, **Figure 3B**), when compared to the vehicle.

To assess the involvement of the supraspinal pool of GPER in the action of ERα17p, 5 µg/mouse of G-15 were injected i.c.v., 20 min before a systemic injection of the peptide (2.5 mg/kg, i.p.) or of the vehicle (10 ml/kg). Except for an isolated peak at 45 min, the scores obtained in animals treated with ERα17p and pre-treated with G-15 were not different from those of the vehicle group throughout the experiment (**Figure 3C**). PWT AUC values confirmed the anti-hyperalgesic properties of the peptide (AUC ERα17p alone: 81.29 ± 8.44 g.min.; AUC vehicle: 7.51 ± 5.35 g.min., *p* > 0.001, Kruskal-Wallis test) and the marked decrease in its effect by G-15 (**Figure 3D**), revealing, thereby, that the anti-hyperalgesic effect of systemic ERα17p involves supraspinal GPER.

**Figure 4:**
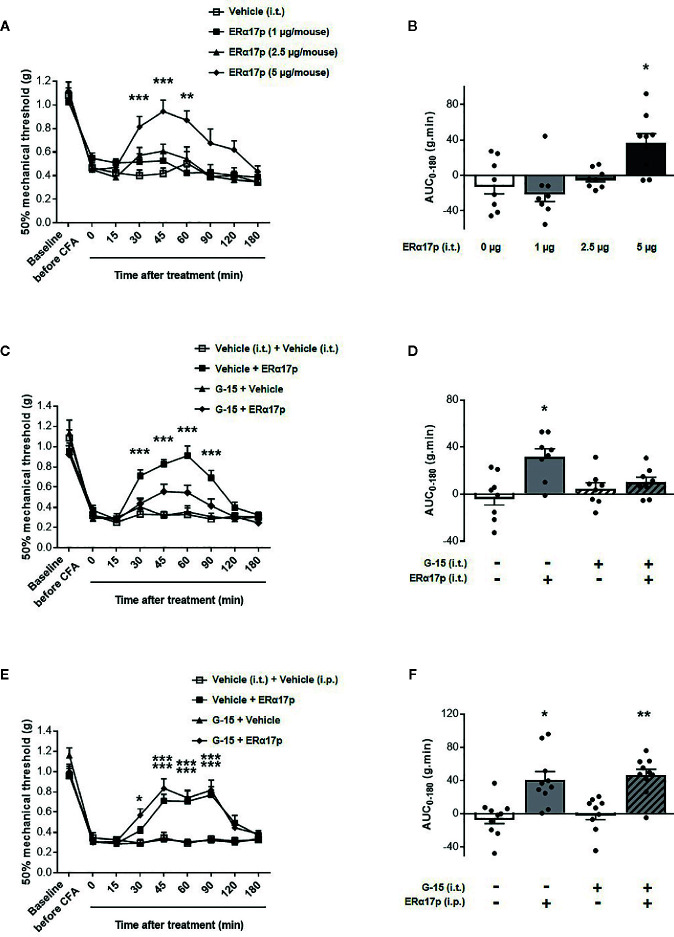
Spinal GPER is not involved in the action of ERα17p in the CFA model. **(A)** Time-course effect of the intrathecal administration of vehicle (saline solution, reference, 2 µl), ERα17p (1, 2.5 and 5 µg/mice) on mechanical hypersensitivity in CFA mice. **(C)** Evaluation of the effect of intrathecally administered ERα17p (5 µg/mice) or vehicle 20 min after G-15 (5 µg/mice, i.t.) or vehicle administration. **(E)** The involvement of spinal GPER in the mechanism of action of systemic ERα17p is investigated by testing ERα17p i.p. with or without G-15 i.t. Mice were i.t. pre-treated with vehicle (saline solution, 2 µl/mice, reference) or G-15 (5 µg/mice) 20 min before an administration of vehicle (saline solution, reference) or ERα17p (2.5 mg/kg, i.p.). **(B, D, F)** Area under the time-course (AUC, 0–180 min) of PWT variations from **(A, C, E)**, respectively. Data are expressed as mean ± SEM (n = 8–9 *per* group). *p < 0.05, **p < 0.01, ***p < 0.001, compared with the vehicle group; two-way ANOVA followed by Dunnett *post hoc* test for time comparison or Kruskal-Wallis test for AUC mean comparison.

The effects of the peptide at the spinal level were extensively studied with the same strategy (**Figure 4**). Intrathecally administered ERα17p at doses of 1, 2.5 and 5 µg/mouse induced a significant anti-hyperalgesic effect compared to vehicle, only at the dose of 5 µg/mouse at times 30 min (ERα17p: 0.81 ± 0.08 g; vehicle: 0.40 ± 0.05 g, *p* < 0.001, Dunnett *post-hoc* test), 45 min (ERα17p: 0.94 ± 0.10 g; vehicle: 0.45 ± 0.05 g, *p* < 0.001, Dunnett *post-hoc* test) and 60 min (ERα17p: 0.87 ± 0.10 g; vehicle: 0.50 ± 0.14 g, *p* = 0.004, Dunnett *post-hoc* test) (**Figure 4A**). This observation was confirmed by AUC (**Figure 4B**). The anti-hyperalgesic effect of 5 µg/mouse of ERα17p (i.t.) was reduced by G-15 (5 µg) co-administered 20 min before by the same route (**Figures 4C, D**). Thus, the anti-hyperalgesic effect of ERα17p directly administered in the spinal cord is mediated by GPER.

The fact that a drug involves a local target when injected locally does not mean that it is the case when it is systemically administered. We therefore investigated the involvement of spinal GPER after an intraperitoneal injection of ERα17p. When intrathecally injected 20 min before a systemic injection of ERα17p (2.5 mg/kg, i.p.), G-15 (5 µg/mouse) failed to modify the anti-hyperalgesic action of the peptide (**Figure 4E**), as confirmed by AUCs. Indeed, both AUC of ERα17p (40.73 ± 10.0 g.min.) and AUC of ERα17p+G15 (46.55 ± 7.12 g.min.) were significantly increased compared to AUC of vehicle (−4.63 ± 7.08 g.min., *p* = 0.014 and *p* = 0.001, Kruskal-Wallis test, respectively) but not statistically different between them *p* > 0.999, Kruskal-Wallis test (**Figure 4F**). This result indicates that the anti-hyperalgesic effect of systemic ERα17p is not mediated by spinal GPER.

### ERα17p Exerts GPER-Dependent Anti-Inflammatory Effects

**Figure 5:**
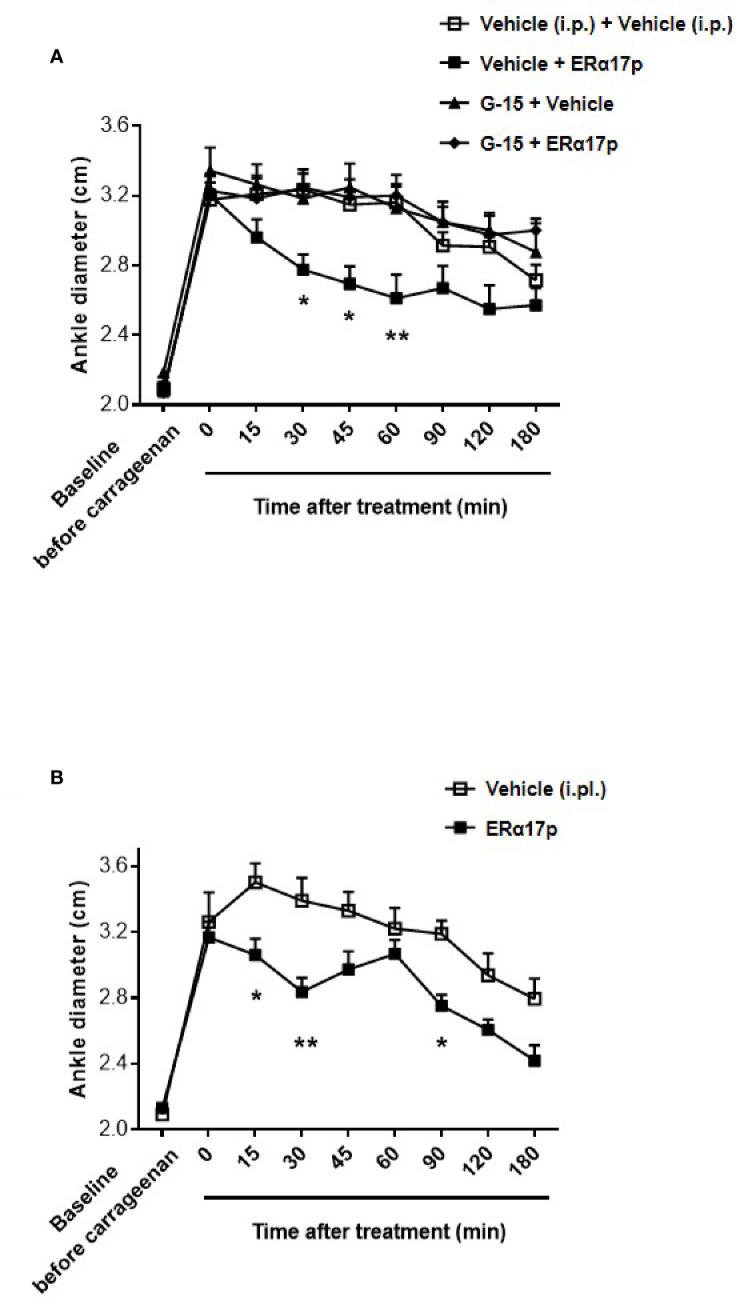
GPER involvement in the anti-inflammatory action of ERα17p in the carrageenan model. **(A)** Ankle diameter of mice was measured before (baseline) and 4 h after carrageenan injection. The involvement of GPER in the mechanism of action of ERα17p was investigated with or without G-15. Mice were i.p. pretreated with vehicle (5% DMSO, 5% Tween80 in saline solution, 10 ml/kg, reference) or G-15 (0.3 mg/kg), 20 min before the administration of vehicle (saline solution, reference) or ERα17p (2.5 mg/kg, 10 ml/kg, i.p.). **(B)** Effect of an intra-plantar (i.pl.) injection of vehicle (saline solution, 10 µl, reference) or of ERα17p (20 µg) on edema measured by ankle diameter (in cm) induced by carrageenan. Data are expressed as mean ± SEM (n = 10–12 *per* group). Two-way ANOVA followed by Dunnett *post hoc* test **(A)** or Sidak *post hoc* test **(B)**. *p < 0.05, **p < 0.01 compared with the vehicle group.

The anti-inflammatory action of ERα17p was explored by measuring its impact on carrageenan-induced edema. Four hours after an intraplantar (i.pl.) carrageenan injection, the diameter of the paw significantly increased from 2.10 ± 0.03 cm to 3.24 ± 0.05 cm (n = 43, *p* < 0.001, *t*-test; **Figure 5A**). After a systemic administration of ERα17p (2.5 mg/kg, i.p.), the time-course of the ankle diameter showed reduced edema from 30 min (ERα17p: 2.78 ± 0.9 cm; vehicle: 3.24 ± 0.09 cm, *p* = 0.03, Dunnett *post-hoc* test, **Figure 5A**) to 60 min (ERα17p: 2.61 ± 0.14 cm; vehicle: 3.16 ± 0.11 cm, *p* = 0.03, Dunnett *post-hoc* test). A pre-treatment with G-15 (0.3 mg/kg, i.p.) 15 min before the injection of ERα17p (2.5 mg/kg, i.p.), abolished the previously observed anti-inflammatory action of the peptide (**Figure 5A**).

In the last part of this work, we investigated a potential local anti-inflammatory action of ERα17p. The peptide was directly administered in the paw at a concentration close to the highest soluble dose (*i.e*., 20 µg in 10 µl *per* mouse, i.pl.). We observed a significant decrease in carrageenan-induced edema (*i.e.*, ankle diameter) at 15, 30, and 90 min (**Figure 5B**). The maximum effect was observed 30 min after the injection (ERα17p: 2.84 ± 0.09 cm; vehicle: 3.39 ± 0.14 cm, *p* = 0.002, Sidak test, **Figure 5B**).

## Discussion

Several studies that have outlined the involvement of the hepta-transmembrane estrogen receptor GPER in pain (8, 29–33) have prompted our interest in studying the influence of the GPER inverse agonist ERα17p (15) on hyperalgesia. We were all the more interested in this approach that a number of ER ligands have been shown to be involved in nociceptive responses including those responses resulting from rheumatoid arthritis (50–52).

In the present study, we have shown that CFA-induced hypersensitivity was markedly reduced by one i.p. injection of ERα17p at a concentration of 2.5 mg/kg, which is the concentration required to achieve maximum effect. The results obtained with 2.5 and 10 mg/kg ERα17p are similar , suggesting a saturation of the signaling cascade or the formation of pharmacologically inert peptide aggregates. Indeed, it has been shown that ERα17p was prone to form amyloid-like fibrils and aggregates *in vitro* (53, 28). Although internalized in vacuoles, these fibrils and aggregates are devoid of cytotoxicity (28).

We then sought to identify the receptor by which ERα17p could exert supraspinal analgesia. GPER is expressed all along pain pathways (4, 6, 7, 54) and is involved in pain modulation (5). Accordingly, G-1, a specific GPER agonist belonging to the family of the cyclopentyl[c]quinolines, induces nociception when systemically (33, 55) or locally (8, 9, 29) administered. Likewise, tamoxifen and fulvestrant, two GPER agonists, induce painful symptoms (56–60). Fulvestrant induces painful disorders such as headache and joint and musculoskeletal pain (61, 62).

Since we have previously demonstrated that the anti-proliferative activity of ERα17p was mediated through the GPER (15), we have hypothesized that this membrane receptor could constitute the keystone of the anti-hyperalgesic action of the peptide. Accordingly, we have observed that the anti-hyperalgesic action of ERα17p was abrogated by the GPER antagonist G-15, highlighting a GPER-dependent mechanism. As reported by others, G-15 fails to influence by itself pain threshold (PWT) when systemically administered in inflammatory and neuropathic models (63), or when intrathecally injected in a neuropathic model (64). The absence of G-15-mediated analgesic effects, whereas the GPER inverse agonist ERα17p is active, reinforces the concept of an intrinsic/constitutive physiological pro-nociceptive profile of GPER.

We then assessed the ability of ERα17p to cross the blood brain barrier. Using a Cy5-labeled (fluorescent) version of the peptide, we observed a strong fluorescence signal at the spinal cord and in the brain. Since cyanines, *per se*, do not diffuse in the CNS (65, 66), we assume that the brain and spinal cord staining detected with H_2_N-ERα17p-Pra(Cy5)-COOH would be exclusively due to the peptide, which consequently is able to cross the blood-brain barrier. The mechanism by which ERα17p reaches the CNS will be subject to future investigations.

Analgesic activity was observed following direct injection of the peptide into the brain. Although this suggests that the brain could be the site of action of the peptide, it does not necessarily imply a direct involvement of a supraspinal GPER population. Thus, we administered ERα17p intraperitoneally and G-15 *via* the intracerebroventricular route. In these experimental conditions, G-15 did not affect pain threshold on its own but decreased ERα17p-induced analgesia, thus definitively confirming the involvement of a supraspinal pool of GPER.

An anti-hyperalgesic effect GPER-dependent was also observed when the peptide was injected intrathecally. Opinion differs greatly on the involvement of the spinal cord pool of GPER in nociception, with some authors providing evidence of the nociceptive effects of G-1 when intrathecally injected (9) and others failing to detect any effect (32, 64, 67). Despite its ability to diffuse into the spinal cord, analgesic effects resulting from a systemic administration of ERα17p were not abolished by an intrathecal injection of G-15. Thus, a spinal action of the systemically administered peptide in the spinal cord seems unlikely. This apparent discrepancy could be due to the fact that the spinal concentration of the peptide after its systemic administration of the would be peptide, too small to induce analgesic effect.

Finally, we observed that ERα17p possessed an anti-inflammatory effect at the dose of 2.5 mg/kg. This effect being fully abolished by G-15, a GPER-induced pro-inflammatory constitutive activity is likely (8, 29–33). Some studies show no evidence of the beneficial effects of pure GPER agonists on inflammation (63, 68, 69) while others have concluded to their anti-inflammatory action (34, 38, 39). Although further investigations are required the concomitant anti-hyperalgesic and anti-inflammatory effects displayed by ERα17p strongly suggest that this peptide could be of clinical interest in the management of inflammatory pain.

## Conclusion

By using mouse models of mechanical hypersensitivity and inflammation, we have shown that the GPER inverse agonist and antitumor compound ERα17p was active *in vivo* on pain and inflammation. These effects were observed at the dose of 2.5 mg/kg and upward, i.e., at doses for which antitumor activity is also observed. These beneficial effects were abolished by the specific GPER antagonist G-15, leading to the conclusion that is involved. We have also evidenced that the anti-hyperalgesic action of ERα17p occurred at the supraspinal level. The mechanism by which the peptide cross the blood brain barrier remains to be determined. Finally, our results suggest that peptides resulting from the proteasome-dependent ERα turnover could play a pivotal role in some physiological and pathological processes through the GPER membrane protein (70).

## Data Availability Statement

The raw data supporting the conclusions of this article will be made available by the authors, without undue reservation.

## Ethics Statement

The studies involving animals were reviewed and approved by the Auvergne Animal Experiment Ethics Committee, CE2A and by the French Ministry of Higher Education and Innovation (authorization N° 18022) and performed according to European legislation (Directive 2010/63/EU) on the protection of animals used for scientific purposes, and complied with the recommendations of the International Association for the Study of Pain.

## Author Contributions

YJ, AE, and CM conceived the design of this study. LB, SL, CC, and CM performed experiments. LB, CC, CM, AE, and YJ analyzed and interpreted data. YJ, CM, and AE wrote the manuscript. All authors contributed to the article and approved the submitted version.

## Funding

This work was supported by CNRS, INSERM, Université Clermont Auvergne, and Université de Paris (Paris Descartes). The authors acknowledge the support received from the Agence Nationale de la Recherche (ANR) of the French government through the program “Investissements d’Avenir” (I-Site CAP 20-25).

## Conflict of Interest

The authors declare that the research was conducted in the absence of any commercial or financial relationships that could be construed as a potential conflict of interest.
