# Supplementary material for: The Antitumor Peptide ERα17p Exerts Anti-Hyperalgesic and Anti-Inflammatory Actions Through GPER in Mice
Source: Front Endocrinol (Lausanne). 2021 Mar 17;12:578250. doi: 10.3389/fendo.2021.578250 (PMC8011567; doi:10.3389/fendo.2021.578250)
Supplement: Supplementary file 1 [file DataSheet_1.docx]

Supplementary Material

# Supplementary Material and Methods

**Acetic-acid-induced writhing test**

This test involves the intraperitoneal administration of 0.6% acetic acid (10 mL/kg) in mice. Following administration, the animals were placed in a polyethylene box, and the frequency of writhing was counted for 10 min. Writhing was characterized by the extension of the lower limbs and elongation of the body. Drugs were administered 30 min before the test.

**Spontaneous locomotor activity**

Spontaneous locomotor was computer analyzed over a 15 minutes period, and the travelled distance was determined. Drugs were administered 15 min before the test.

# Supplementary Figure

**Supplementary Figure 1: ERα17p induced a reduction of writhing in the acetic acid test without impairing spontaneous activity.** (**A**) Number of writhing induced by intraperitoneal acetic acid (0.6%) injection were counted after i.p. treatment with vehicle (saline solution, 10 mL/kg), ERα17p (1.25, 2.5 or 10 mg/kg) or morphine (1 mg/kg). (**B**) Spontaneous locomotion during 15 minutes after i.p. treatment with vehicle (saline solution, 10 mL/kg) or ERα17p (1.25, 2.5 or 10 mg/kg). Data are shown as mean ± SEM (n = 6-8 *per* group). Kruskal-Wallis test was performed. **p* < 0.05, ***p* < 0.01, ****p* < 0.001 compared with the vehicle group.
